# Complimentary Methods for Multivariate Genome-Wide Association Study Identify New Susceptibility Genes for Blood Cell Traits

**DOI:** 10.3389/fgene.2019.00334

**Published:** 2019-04-26

**Authors:** Segun Fatumo, Tommy Carstensen, Oyekanmi Nashiru, Deepti Gurdasani, Manjinder Sandhu, Pontiano Kaleebu

**Affiliations:** ^1^Uganda Medical Informatics Centre, MRC/UVRI and LSHTM Uganda Research Unit, Entebbe, Uganda; ^2^London School of Hygiene and Tropical Medicine, London, United Kingdom; ^3^H3Africa Bioinformatics Network (H3ABioNet) Node, Centre for Genomics Research and Innovation, NABDA/FMST, Abuja, Nigeria; ^4^Human Genetics, Wellcome Sanger Institute, Hinxton, Cambridge, United Kingdom; ^5^Division of Computational Medicine, Department of Medicine, University of Cambridge, Cambridge, United Kingdom

**Keywords:** multivariate GWAS, PCA, full blood counts, multiple phenotype, genome-wide association study

## Abstract

Genome-wide association studies (GWAS) have found hundreds of novel loci associated with full blood count (FBC) phenotypes. However, most of these studies were performed in a single phenotype framework without putting into consideration the clinical relatedness among traits. In this work, in addition to the standard univariate GWAS, we also use two different multivariate methods to perform the first multiple traits GWAS of FBC traits in ∼7000 individuals from the Ugandan General Population Cohort (GPC). We started by performing the standard univariate GWAS approach. We then performed our first multivariate method, in this approach, we tested for marker associations with 15 FBC traits simultaneously in a multivariate mixed model implemented in GEMMA while accounting for the relatedness of individuals and pedigree structures, as well as population substructure. In this analysis, we provide a framework for the combination of multiple phenotypes in multivariate GWAS analysis and show evidence of multi-collinearity whenever the correlation between traits exceeds the correlation coefficient threshold of *r*^2^ >=0.75. This approach identifies two known and one novel loci. In the second multivariate method, we applied principal component analysis (PCA) to the same 15 correlated FBC traits. We then tested for marker associations with each PC in univariate linear mixed models implemented in GEMMA. We show that the FBC composite phenotype as assessed by each PC expresses information that is not completely encapsulated by the individual FBC traits, as this approach identifies three known and five novel loci that were not identified using both the standard univariate and multivariate GWAS methods. Across both multivariate methods, we identified six novel loci. As a proof of concept, both multivariate methods also identified known loci, *HBB* and *ITFG3*. The two multivariate methods show that multivariate genotype-phenotype methods increase power and identify novel genotype-phenotype associations not found with the standard univariate GWAS in the same dataset.

## Introduction

Genome-wide association studies (GWAS) have discovered loci associated with a extensive range of human traits and diseases. Mostly, the standard univariate GWAS approach has been performed in a single trait framework without putting into consideration clinical relatedness and correlations among phenotypes. However, as many human traits are highly correlated, given the usual stringent statistical genome-wide significance threshold, such analyses may have a number of limitations including difficulties in identifying genetic risk factors implicating pleiotropic effects (Park et al., 2011). Current large-scale standard univariate and multivariate GWAS analyses have principally concentrated on the populations of European lineage (Need and Goldstein, 2009; Zhang et al., 2009; Galesloot et al., 2014; Porter and O’Reilly, 2017) with only a few small-scale GWAS in African populations across a narrow range of cardiometabolic diseases and traits (Gurdasani et al., 2015; Peprah et al., 2015). In order to generalize the discoveries from genetic studies of complex diseases and provide opportunities for new understandings into disease etiology and potential therapeutic strategies, it will be vital to investigate the genetic susceptibility in a global setting, including populations of African ancestry (McCarthy et al., 2008; Adoga et al., 2014).

Multivariate linear mixed models have been extensively used in a range of genetics studies (Yu et al., 2006; Kang et al., 2008, 2010; Zhang et al., 2010; Lippert et al., 2011; Loh et al., 2015; Hackinger and Zeggini, 2017). Recently this approach has attracted substantial topical interest in GWAS. Genome-wide Efficient Mixed Model Association (GEMMA) (Zhou and Stephens, 2014) models a multivariate linear mixed model to test SNPs associations with multiple traits simultaneously while adjusting for population stratification. In previous studies, multivariate analyses have mainly been performed on GWAS of lipids traits (Park et al., 2011) and anthropometry traits (Ried et al., 2016) mostly in the European and Asian populations. As cellular components of the full blood count (FBC) arise from a common pluripotent stem cell (Seet et al., 2017) and are highly correlated. Thus, FBC traits provide an opportunity to: (1) explore how multivariate GWAS performs in comparison with standard univariate analyses in a family-based dataset, (2) investigate the effect of highly correlated traits in multivariate analyses, (3) explore different multivariate approaches in GWAS, and (4) understand when a multivariate analysis would be most helpful in a GWA study. In the present study, we performed the first multivariate GWAS of FBC traits by analyzing quality controlled 2,230,258 autosomal SNPs in nearly 7000 individuals who are structured in clustered groups in rural Uganda, genotyped on the Illumina Human Omni 2.5 M octo array. We applied a two way complementary multivariate GWAS strategies in nearly 5000 genotyped samples and validation of the associated genetic variants in ∼2000 individuals with whole genome sequencing (WGS) sampled from Ugandan General Population Cohort (GPC).

## Materials and Methods

### Study Population

**FIGURE 1:**
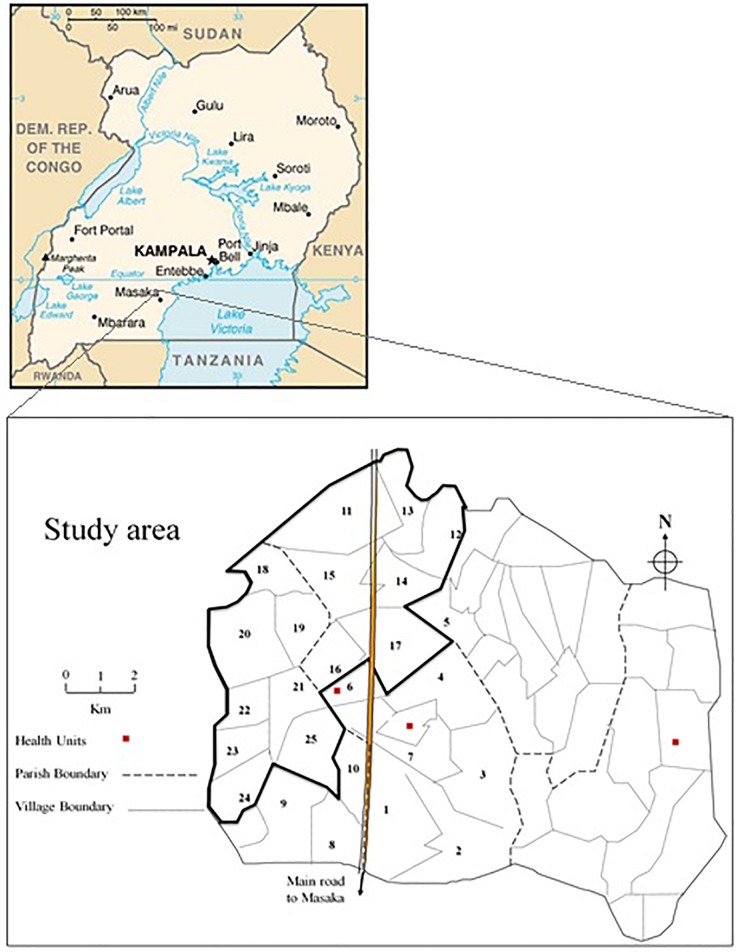
Study area in Kyamulibwa sub-region.

General Population Cohort is a population-based open cohort of roughly 22,000 inhabitants around 25 neighboring villages of Kyamulibwa, which is a subcounty of Kalungu district in countryside south-west of Uganda (Figure 1). The cohort study was founded in the late 80s by the Medical Research Council (MRC) United Kingdom in partnership with the Uganda Virus Research Institute (UVRI) to primarily investigate the trends in incidence and prevalence of HIV infection in Uganda. Samples were collected from research participants during a survey from the research study area. The study area is clustered into villages defined by governmental borders ranging in size from 300 to 1500 dwellers and includes numerous families resident within households (Asiki et al., 2013). The GPC Round 22 study took place in 2011 through collaboration between the University of Cambridge, Wellcome Sanger Institute (WSI), and MRC/UVRI. The study was contained within one annual survey round of the longitudinal cohort. The focus of the GPC Round 22 study was to investigate the genetics and epidemiology of communicable and non-communicable diseases to provide etiological insights into the genetic variation in cardiometabolic and infectious risk factors in children and adults using both population genetic and epidemiological approaches. The first set of samples tagged UGWAS was constituted of ∼5000 Uganda subjects genotyped on the HumanOmni2.5-8 Illumina genotyping chip array. Following a stringent quality control (see section “Quality Control”), 4778 individuals were carried further for analysis. The later set of samples tagged UG2G were ∼2000 individuals who underwent whole genome sequencing, of these 1,629 individuals passed quality checks and were non-overlapping with the genotype data. Both UGWAS and UG2G included several pedigrees, and individuals with cryptic relatedness, as well as individuals clustered by household and village. Due to extensive migration into and around the region, nine ethno linguistic groups in south-western Uganda were included in the sample.

### Study Design

The data collection of GPC Round 22 study contained five main stages which took place in 2011 over the course of the year: mobilization (recruitment and consenting), mapping, census, survey, and feedback of results and clinical follow-up. The census consisted of a family questionnaire and questionnaire for the individual recruited from within the family. The family questionnaire was completed by the head of family or another responsible adult or emancipated minor member of the household. The household census questionnaire focused on sociodemographic information about the household, such as the quality of the house, property ownership, and employment of workers. The individual survey questionnaire captured information on members of a household including position within household, marital status, resident status, childbirth, and fertility, tribe, and religion. Information on lifestyle and health was obtained using a standard questionnaire. This included biophysical measurements and blood samples (Asiki et al., 2013). To assess the spectrum of genetic variants associated with cardiometabolic traits in this population, we previously performed a standard univariate GWAS in a range of individual cardiometabolic traits. In the current study, we applied two different multivariate GWAS methods in analyzing multiple related FBC phenotypes simultaneously following a standard univariate GWAS analysis of the individual trait. We assessed the autosomal common SNPs in the imputed genotyped data (UGWAS) and sequenced UG2G in a pooled analyses comprising of 6407 all individuals, rather than a meta-analysis which would consider these as independent datasets and potentially result in inflation of type I error.

### Quality Control

Briefly, we applied stringent quality control filtering to carry out a succession of sequential quality control steps on ∼5000 Uganda samples genotyped on an Illumina array. Specifically, a total of 2,314,174 autosomal variants were genotyped on the illumine HumanOmni2.5-8 array. We excluded 39,368 autosomal variants who did not pass the stringent quality control cutoff for the variants (Heckerman et al., 2016). We also excluded a total of 91 individuals during sample QC as they fail to meet the quality control cutoffs for the samples call rate (>97%) or for the heterozygosity in the range of mean ± 3SD, or because they fail the gender check criteria using the X-chromosome as a match. Three samples were also excluded because of they are too related to one another using identical by descent (IBD >0.90) (Heckerman et al., 2016). Downstream analyses were performed on the remaining 2,230,258 autosomal markers and 4,778 samples which passed quality checks. The workflow for data processing of UG2G has been previously described in more detail.

### Genotype Imputation

Imputation was carried out on pre-phased data with IMPUTE2 (Howie et al., 2009) using a merged reference panel of the whole genome sequence data from the African Genome Variation Project (Gurdasani et al., 2015), the UG2G described earlier and the 1000 Genomes phase 3 project (1000 Genomes Project Consortium, 2015) following standard recommendations. Imputation was carried out in chunks of two MB and then concatenated. In order to allow the most accurate different downstream analyses, imputed SNPs were further filtered at info statistics of 0.3 and a minor allele frequency (MAF) threshold of 0.5%. All duplicated sites and variants were also removed from the data. Analyses were carried out on the final set of 18,868,552 QC imputed data. This approach removed all monomorphic variants from the data which is based on Genome Research Consortium human build 37 also called the Human genome build 19.

### Phenotype Definition and Transformation

Fifteen FBC traits were measured using the Beckman Coulter ACT5 Diff CP hematology analyzer (Table 1). We carried out the inverse normal transformation of each trait residual. First, we obtained residuals after the regression of each trait on age, age2, and sex. We then inverse normally transformed the residuals for GWAS analysis.

**Table 1:** A description of phenotypic traits analyzed in the total 6407 individuals in the pooled dataset.

Traits	Unit	Number of analyzed samples	Mean	Standard Deviation
White blood cell (WBC)	×10^9^/l	1625	5.17	1.52
Red blood cell (RBC)	×10^9^/l	1625	4.72	0.61
Mean corpuscular volume (MCV)	fl	1625	85.9	7.77
Mean cell hemoglobin (MCH)	pg/cell	1625	28.9	2.93
Mean cell hemoglobin concentration (MCHC)	g/L	1625	33.63	1.18
Red blood cell distribution width (RDW)	%	1625	13.11	1.37
Packed cell volume (PCV)	l/l	1625	40.31	4.61
Hemoglobin	g/L	1625	13.56	1.6
Mean platelet volume (MPV)	fL	1624	8.65	0.81
Platelet count (PLT)	x10^9^/l	1625	219	79.1
Lymphocyte count	%	1565	48.64	9.9
Monocyte count	%	1565	5.65	1.93
Basophil count	%	1565	0.91	1.03
Neutrophil count	%	1415	38.02	10.5
Eosinophil count	%	1555	6.73	6.50

### Evaluation for Systematic Difference Between Genotype and Sequence Data

**FIGURE 2:**
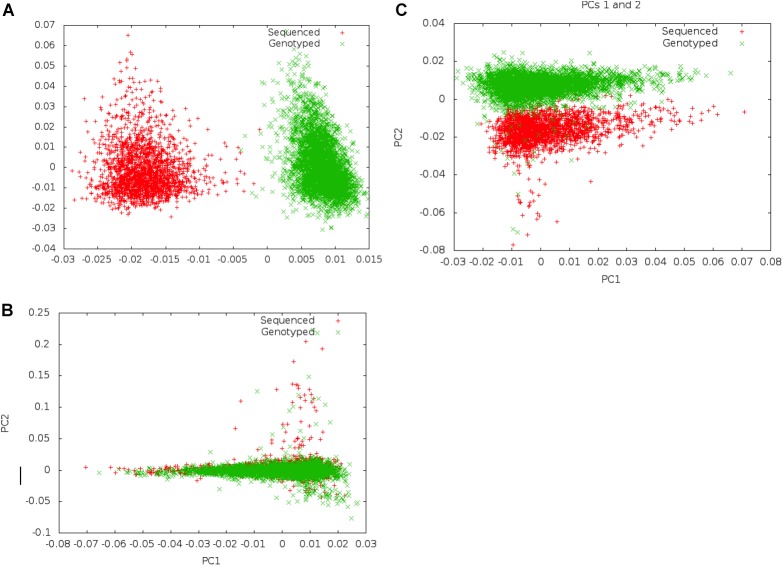
Separation on PCA between genotype and Sequence data using different filters. Panel **(A)** shows separation of sequenced and imputed genotyped samples in pooled PCA analysis of 6,407 individuals from UGR along PC 1. On filtering of variants that showed <80% concordance between sequenced and genotype data in a set of 343 individuals who were genotyped and sequenced, this separation was reduced, and became a secondary axis of variation (along PC 2) **(B)**. Panel **(C)** shows that separation along PCs 1 and 2 is removed on filtering variants with concordance <90% between sequenced and genotyped data.

Following merger of imputed genotype and sequence data, we first examined if systematic differences existed between imputed genotype data and sequence data (Figure 2). We carried out principal component analysis (PCA) on these data to examine whether there was separation by data mode (imputed genotype data and sequenced data). We noted clear separation of data points of genotype imputed and sequence data on PCA. In order to minimize systematic effects, we examined the 343 samples that had been genotyped and sequenced in duplicate. Using these samples, we evaluated different thresholds of concordance between sequence and imputed genotype data for identical samples, filtering out SNPs that showed a concordance <0.80 and <0.90, in the 343 samples. We found that a minimum concordance threshold of 0.90 was required to abolish systematic effects observed between genotype array and sequence data on PCA.

Following exclusion of 904,283 variants (2.3% of all variants) that showed <90% concordance in genotypes between the sequence and imputed genotype data (for 343 samples that had been genotyped and sequence), PCAs did not show any systematic differences between imputed genotype and sequence data. We inspected the first ten PCs to ensure that systematic differences did not represent an important axis of variation in the genetic data. Following filtering, a total of 39,312,112 autosomal markers in the joint set of 6,407 samples were taken forward for analyses. For GWAS association analyses, we only included a subset of variants (*n* = 20,594,556) that met an MAF threshold of at least 0.5%.

### Statistical Methods for Association Analysis

We used the exact linear mixed model approach implemented in GEMMA version 24 for analysis of pooled data from 6,407 individuals in GPC. We evaluated different approaches for generation of the kinship matrix to control type I error in analysis. It has been shown that inclusion of causal SNPs in the kinship matrix can lead to overly conservative results for these SNPs, and reduction in power for GWAS discovery. In order to maximize discovery, we used the leave one chromosome out (LOCO) approach for analysis (Listgarten et al., 2012; Yang et al., 2014). In this approach each chromosome is excluded from generation of the kinship matrix in turn, for association analysis for markers along that chromosome. This ensures that causal SNPs at a locus on a given chromosome are not used for generation of the kinship matrix used in analysis of that specific chromosome. Therefore, we generated 22 kinship matrices for analysis, each excluding the chromosome being analyzed using the given matrix.

For computational efficiency, and to avoid correlation effects due to LD, we LD pruned the data prior to calculation of the GRM matrix for each LOCO analysis. We carried out sensitivity analyses using different r2 thresholds for pruning, to examine whether type I error was appropriately controlled on examining genomic inflation factors from QQ plots. We finally used all markers with an MAF >1%, pruned to an r2 threshold of 0.5, using PLINK (Purcell et al., 2007) with the flags –maf 0.05 and –indep-pairwise 100 10 0.5, where 0.01 is the minimum MAF threshold of 1% and 0.5 is the r2 threshold within each 100 marker window sliding by a step size of 10 markers during each iteration. All genomic inflation factors for traits were noted to be below 1.05 using this approach.

We also included a covariate to indicate whether data originated from imputed genotyped individuals or sequenced individuals to allow for any systematic differences between data (although earlier PCA suggested no systematic effects in filtered data). A MAF threshold of 0.5% was applied in GEMMA analysis. The 20674434 variants that passed all quality control (QC) criteria were tested for associations using the standard univariate (UV-GWAS), multivariate approach (MV-GWAS) and principal component approach (PC-GWAS). These methods were described in the Sub-sections “Univariate GWAS Method (UV-GWAS), Multivariate GWAS Method (MV-GWAS), and Principal Component GWAS Method (PC-GWAS).” For each analysis, the *P*-values were calculated using the likelihood ratio test.

#### Univariate GWAS Method (UV-GWAS)

Here, we carried out a genome wide association study of 15 FBC traits (Table 1) using the standard univariate approach. We examined the association between a single trait at a time with SNPs taking into consideration issues with relatedness and population stratification. We show the distribution of association *P*-values for the 15 traits in QQ plots (Supplementary Figure 1). The genomic inflation factor for each analysis ranges from between 0.99 and 1.01 suggesting there is no genome-wide inflation due to population stratification. We show a summary of all genome-wide significant variants in Table 2.

**Table 2:** Description of genome-wide significant loci using the standard univariate GWAS approach.

Peak SNP	AF	Association count	Chr	BP (GRCh37)	Gene	Trait	Pooled analysis *P*-value
rs334	0.923	46	11	5248232	*HBB*^∗^	RDW	5.56E-17
rs13331259	0.897	230	16	299923	*ITFG3*^∗^	RBC, MCV, MCH, MCHC	1.23E-30
rs1347767	0.152	277	2	136485657	*R3HDM1*^∗^	Neutrophil count	7.81E-12
19:16213697	0.862	4	19	16213697	*TPM4*^∗^	MPV	4.62E-10
rs12534473	0.502	1	7	106374548	*CTB-30L5.1*	MPV	2.00E-08
rs7725036	0.742	1	5	97438982	*AC008834.1*	RBC	4.71E-08
rs142586351	0.9	1	19	21800425	–	Eosinophil count	2.78E-08
rs2769976	0.909	1	20	48514919	–	Eosinophil count	5.90E-09

*^∗^Known association; AF, allele frequency.*

#### Multivariate GWAS Method (MV-GWAS)

**FIGURE 3:**
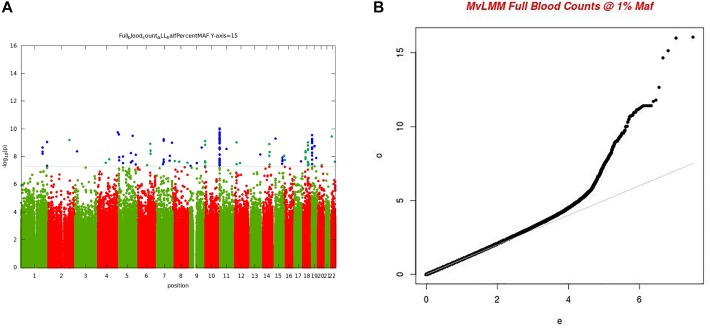
**(A)** Manhattan plot of genome-wide associations for FBC (15 full blood count traits). Each point denotes a variant, with the *X*-axis representing the genomic position and *Y*-axis representing the association level -log 10 (*P*-value) for the association of all variants with MAF >=0.005. The dotted line shows the genome-wide significant *P*-value of 5 × 10–8. **(B)** Visually inflated QQ plot of genome-wide associations for FBC (15 full blood count traits) with an inflation factor of 0.97.

**FIGURE 4:**
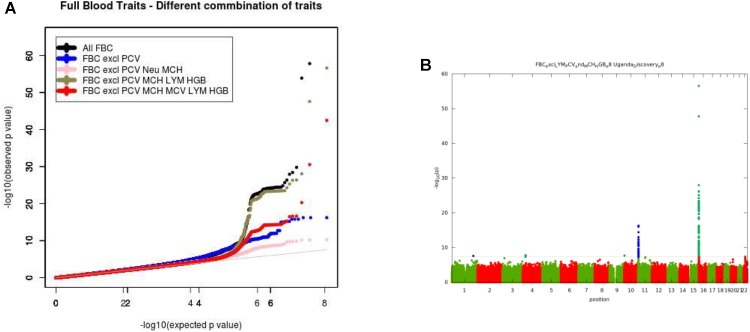
**(A)** QQ plot of genome-wide associations for all FBC excluding highly correlated traits: PCV, MCH, Hgb, and LYM. **(B)** Manhattan plots of genome-wide associations for all FBC after highly correlated traits: PCV, MCH, Hgb, and LYM were excluded from the analysis.

For the multivariate GWAS analysis, we started by testing for marker associations in a multivariate linear mixed model in GEMMA with all the 15 FBC traits simultaneously while we controlled for population stratification without giving consideration to the level of correlations among these traits. We plotted the resulting *P*-values from this association analysis and showed the Manhattan and QQ plots in Figure 3A,B. We noted an unconventional Manhattan plot (Figure 3A) showed genome-wide significant variants at almost every chromosome and the QQ plot showed a lift off from the null line as a visually inflated QQ plot (Figure 3B). Since this could be due to multicollinearity, we calculated the correlation coefficient between all FBC traits (see Figure 4) in order to identify highly collinear variables. Hemoglobin (Hgb) was found to be highly correlated with PCV (*r* = 0.94) and MCH highly correlated with MCV (*r* = 0.92) (full list in Supplementary Tables 1a,b). Repeating the analysis while excluding PCV, MCH, Hgb, and LYM showed an expected QQ plot (Figure 4A) and a conventional Manhattan plot with strong genetics signal at expected chromosomes 11 and 16 (Figure 4B).

In this analysis, we examined multiple correlated traits while taking into consideration issues with relatedness and population stratification. We noted that the issues with multicollinearity that manifest as inflated QQ plots, and unconventional Manhattan plots are particularly due to rare variation. The inflation is mostly for variants with <1% maf, but not all variants causing the inflation are in this category. It seems that rare variants are much more susceptible to unstable estimates with multi-collinearity. This analysis provides a framework for the combination of multiple phenotypes in multivariate GWAS analysis having shown evidence of multi-collinearity whenever the correlation between traits exceeds the correlation coefficient of *r*^2^ >=0.75.

#### Principal Component GWAS Method (PC-GWAS)

Usually, the PCA is an analytic approach used in GWAS for examining population structure, especially within ethno-linguistic groups. Previous studies (Biffi et al., 2010) have used PCs as covariates in their analyses to correct for possible biases induced by sample collection or non-genetic geographical effects on phenotype. However, Ried et al. (2016) effectively applied PCA approach to four correlated anthropometric traits to encapsulate body shape and recommended the approach for other correlated traits such as FBC traits. We explored this approach to complement the standard multivariate GWAS we described in the Section “Multivariate GWAS Method (MV-GWAS).” We applied PCA to the same 15 correlated FBC traits in the same transformed phenotypic dataset to generate a dimensional set of uncorrelated outcome PCs (Supplementary Table 2). We then tested for marker associations with each PC in the univariate linear mixed model in GEMMA. We show that the FBC composite phenotype as assessed by each PC articulates information that is not fully encapsulated by the individual FBC phenotype as this approach identifies genome-wide significant variants that were not identified using both the standard univariate and multivariate GWAS.

#### Significance Thresholds for Multiple Testing

There are many methods such as Bonferroni or Sodak for multiple comparisons tests. These methods exploit the correlation structure between genetic variants to estimate the effective number of independent tests, and then use standard techniques for independent tests to calculate an appropriate significance threshold. In standard univariate GWAS (such as our UV-GWAS), the standard significance threshold of 5 × 10^−8^ is mostly used. For our Mv-GWAS, GEMMA appropriately adjusted for testing multiple phenotypes, so there was no need for an additional correction, however, for PC-GWAS, the Bonferroni correction for testing 15 orthogonal phenotypes obtained from the principal components analysis of the 15 FBC phenotypes (PC-GWAS) would be 5 × 10^−8^/15 (3.33 × 10^−9^). In order to address the potential introduction of type II errors via the application of this rigorous correction, we present all our results using the standard genome-wide significant threshold of *P*-value ≤5 × 10^−8^ was met, but we highlight result with Bonferroni corrected significant threshold.

## Results

For each strategy (UV-GWAS, MV-WAS, PC-GWAS), we applied the typically significance threshold of *p* < 5.0E-08 to define association. We defined a locus to be novel if it had not been associated with any FBC trait in any previous GWAS and its *P*-value is less than or equal to 5 × 10^−8^. In order to define whether a locus was known or novel, we searched the NHGRI database for loci reaching statistical significance at a level 5e-08 associated with FBC trait. This was supplemented by a literature review.

### Results for UV-GWAS

With UV-GWAS method, we analyzed each 15 trait individually and identified 4 novel association signals. This method also confirmed 4 known loci associated with blood traits (Table 2).

#### HBB

We identified important functional variants such as the sickle cell variant (rs334) in the *HBB* gene associated with RDW. The *HBB* locus is found to be associated with RBC distribution width in our main standard univariate analysis. As previously observed in regions affected with malaria, this variant has reached high frequencies as a result of balancing selection because it can provide resistance against the parasite, and reduces the severity of malaria among carriers. This signal was also identified by MV-GWAS and PC-GWAS.

#### ITFG3

We found 230 genome-wide statistical significant variants in the known locus ITFG3 associated with RBC, MCV, MCH, and MCHC. Though the function of *ITFG3* is not known, it is known to be expressed in an erythroleukemia cell line, and other common SNPs of this gene have been implicated with red blood cell indices in European and Asian GWASs (Chen et al., 2013; Hodonsky et al., 2017). This signal was also identified by MV-GWAS and PC-GWAS.

#### R3HDM1

UV-GWAS identified 277 genome-wide statistical significant variants in association signal *R3HDM1* gene on chromosome 2 with neutrophil count; this variant was common in African populations (MAF = 10%), and monomorphic in Europeans. This signal is reported in our study.

#### TPM4

UV-GWAS found 4 genome-wide statistical significant variants in the known locus TPM4. TPM4 plays a crucial role, in association with the troponin complex, in the calcium reliant on regulation of vertebrate striated muscle tightening (Crabos et al., 1991).

#### CTB-30L5.1 and AC008834.1

Both CTB-30L5.1 and AC008834.1 are Uncharacterized, and do not code for protein. CTB-30L5.1 is an RNA Gene which is affiliated with the ncRNA class while AC008834.1 is a processed pseudogene.

### Results for MV-GWAS

Three association loci were identified using the MV-GWAS approach, of which two (*HBB* and *ITFG3*) had been previously reported to be associated with at least one of the FBC traits (Table 3). These known associations were also identified using the standard univariate and PC-GWAS approaches.

**Table 3:** Description of genome-wide significant loci using MV-GWAS.

Peak SNP	AF	Association count	Chr	BP (GRCh37)	Gene	Pooled analysis *P*-value
rs3123543	0.354	1	1	212790686	*ATF3*	1.29E-08
rs334	0.923	56	11	5248232	*HBB*^∗^	5.34E-20
rs13331259	0.897	158	16	299923	*ITFG3*^∗^	7.84E-30

*^∗^Known association; AF, allele frequency.*

#### ATF3

**FIGURE 5:**
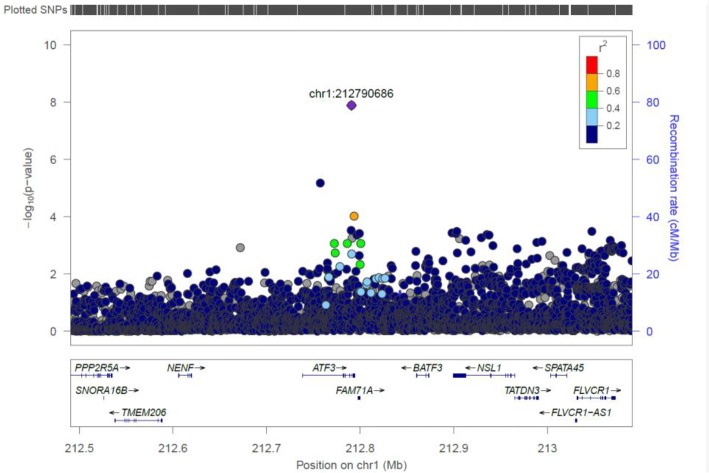
Regional visualization of the GWAS of –log10 of the *P*-value of genomic location ATF3 (rs3123543 in purple), with each dot representing a SNP on the corresponding genes at the bottom.

We identified a common variant rs3123543 association with blood in ATF3 (Figure 5). ATF3 interacts with TP53, JunD proto-oncogene, JUN oncogene, CEBPB, and STAT1, among others. Notably, CEBPB is a vital transcriptional activator in the genes regulation engaged in hemopoiesis and immune and inflammatory responses (Janz et al., 2006).

### Results for PC-GWAS

Five novel association signals were identified using PC-GWAS method (Table 4 and Supplementary Figure 2). It also found two known associations (HBB and ITFG3) that had been previously reported to be associated with at least one of the fifteen FBC traits. These known associations were also identified with UV-GWAS and MV-GWAS approaches and were described in the Sections “*ITFG3* and *R3HDM1*.”

**Table 4:** Genome-wide significant loci using PC-GWAS approach.

Peak SNP	AF	Association count	Chr	BP (GRCh37)	Gene	Pooled analysis *P*-value
rs7296503	0.723	2	12	41700764	PDZRN4^+^	2.10E-09
rs112505971	0.948	21	10	27357470	ANKRD26	1.81E-08
rs4837892	0.652	1	9	124588304	TTLL11	2.99E-08
rs9917425	0.819	4	20	16736045	OTOR^+^	6.77E-10
rs3840870	0.556	1	17	48262183	COL1A1	1.19E-08
rs334	0.923	22	11	5244665	HBB^∗+^	1.15E-12
rs76792961	0.897	209	16	293593	ITFG3^∗+^	6.70E-26
rs2853961	0.603	1	6	31231989	HLA-C^∗+^	4.25E-08

*^∗^Known association; AF, allele frequency. ^+^Locus remain significant after applying Bonferroni corrected threshold of 3.33 × 10^−9^.*

#### PDZRN4

**FIGURE 6:**
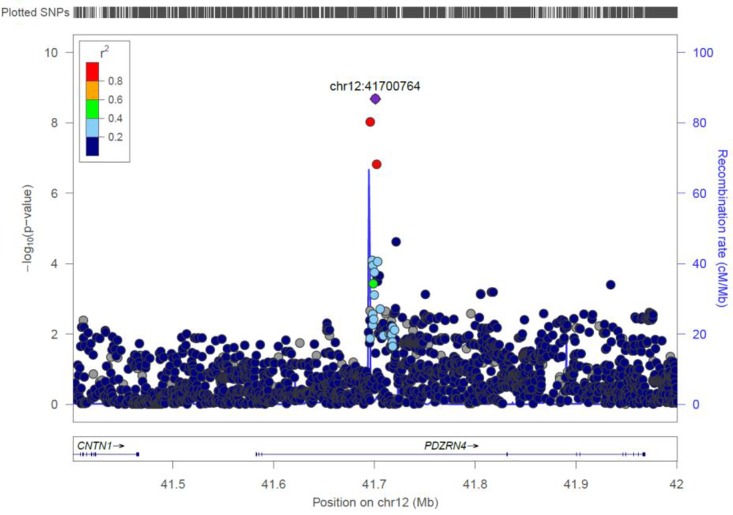
Regional visualization of the GWAS of –log10 of the *P*-value of genomic location PDZRN4 (rs7296503 in purple), with each dot representing a SNP on the corresponding genes at the bottom.

Two genome-wide statistical significant SNPs were identified in PDZRN4 (Figure 6). The locus enlarged epidermal growth factor receptor (EGFR) surface abundance and thus reduced homologous recombination repair frequency, the Negative genetic interaction between MUS81−/− and MUS81+/+, Decreased viability, Increased vaccinia virus (VACV) infection (Sivan et al., 2013) The gene is expressed in the lymph node, colon, bladder, whole blood, among other organs.

#### ANKRD26

**FIGURE 7:**
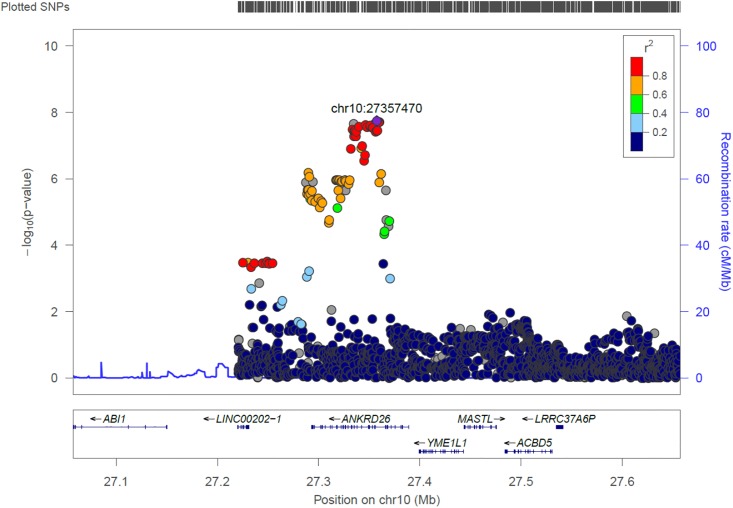
Regional visualization of the GWAS of –log10 of the *P*-value of genomic location ANKRD26 (rs112505971 in purple), with each dot representing a SNP on the corresponding genes at the bottom.

We identified 21 genome-wide association variants at this locus (rs112505971, *P*-value 1.81e-08) (Figure 7). The variant (rs112505971) is common in Ugandan populations, with allele frequency of 0.948. It is noted that the variant is monomorphic in East and South Asian populations but very rare in Ad Mixed American and European populations with maf of 0% in 1000 genomes project. ANKRD26 (Ankyrin Repeat Domain 26) is a Protein-Coding gene. The peak variant is common in Uganda (5%) but absent in EUR and EAS populations. In Clinvar, ANKRD26 is known to be associated Thrombocytopenia 2. This is an autosomal dominant non-syndromic condition which is delineated by reduced numbers of standard platelets, resulting in a moderate bleeding inclination (Pippucci et al., 2011).

#### TTLL11

**FIGURE 8:**
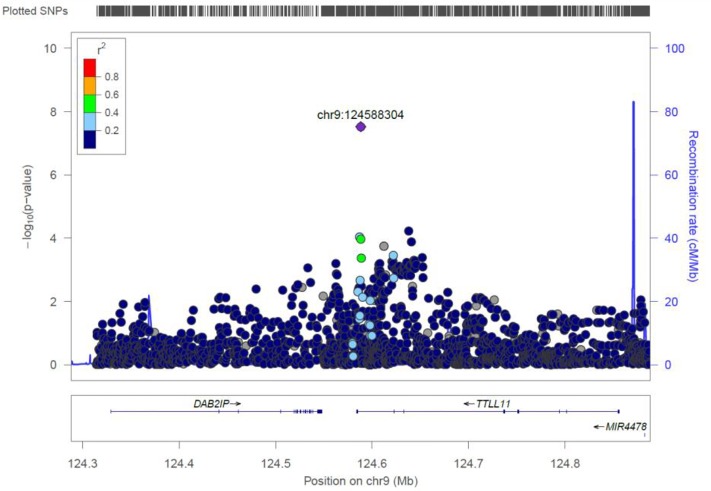
Regional visualization of the GWAS of –log10 of the *P*-value of genomic location TTLL11 (rs4837892 in purple), with each dot representing a SNP on the corresponding genes at the bottom.

rs4837892 in TTLL11 (tubulin tyrosine ligase-like family) is associated with FBC (Figure 8). TTLL11 is expressed in 119 organs including whole blood, white blood cells, lymph node, and cervical spinal cord.

#### OTOR

We identified four novel genome-wide statistical variants at chromosome 20 in the gene OTOR. This gene is known to be associated with posttraumatic stress disorder in GWAS catalog (Xie et al., 2013).

#### COL1A1

One variant was identified in the gene COL1A1 to be associated with blood cell. This gene encodes the pro-alpha1 chains of type I collagen whose triple helix comprises two alpha1 chains and one alpha2 chain. The COL1A1 gene provides instructions for making part of a large molecule called type I collagen.

### Comparison of Genome-Wide Statistical Significant Association Loci Found by UV-GWAS, MV-GWAS, and PC-GWAS

**FIGURE 9:**
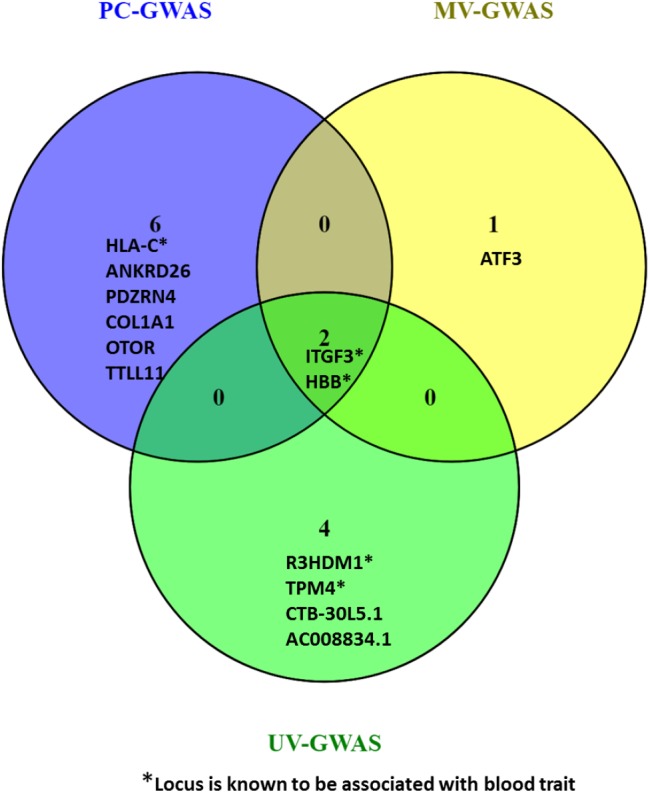
Venn diagram comparing the loci found by PC-GWAS and MV-GWAS in comparison with UV-GWAS. Two known loci HBB and ITFG3 were identified all the three methods. Two novel intergenic variants identified by UV-GWAS not shown.

Collectively, the three methods identified fifteen loci including ten novel loci associated with FBC traits. Two of the novel loci are intergenic variants and not shown in Figure 9.

## Discussion

To assess the spectrum of genetic variants associated with FBC traits in Uganda, we performed standard univariate and two multivariate GWAS methods to examine association of 15 FBC traits in 6407 individuals in a pooled data from UGWAS with UG2G sequence data. Across the three methods, we identified eight novel loci. They include *ATF3* (rs3123543) using MV-GWAS strategy, *PDZRN4* (rs7296503), *ANKRD26* (rs112505971) and *TTLL11* (rs4837892), OTOR (rs9917425), COL1A1 (rs3840870) using PC-GWAS strategy and AC008834.1 (rs7725036), CTB-30L5.1 (rs12534473), two intergenic variants (rs142586351, rs2769976) using UV-GWAS. As a proof of concept, both methods also identified known associated loci *HBB* and *ITFG3*. Additionally, UV-GWAS solely identified an additional variant at known loci TPM4 and *R3HDM1* while PC-GWAS exclusively identified known association locus *HLA-C*. The MV-GWAS has been reported to be especially powerful when the genetic correlation between traits differs from the environmental. I think this effect is not present in the PC-GWAS, because it makes PCs based on phenotypic correlations. Therefore the two methods can be sensitive for different correlation patterns between the traits. The methods complement one another and show also show that multivariate genotype-phenotype methods increase power and identify novel genotype-phenotype associations not found with univariate GWAS in the same dataset.

One limitation of the MV-GWAS approach is its sensitivity to highly correlated traits. Sensitivity analyses showed that issues with multicollinearity may occur, that manifest as inflated QQ plots, and unconventional Manhattan plots particularly due to rare variation using the MV-GWAS strategy. The inflation is mostly for variants with <1% maf, but not all variants causing the inflation are in this category. It seems that rare variants are much more susceptible to unstable estimates with multicollinearity. Evidence of multi-collinearity was seen whenever the correlation between traits exceeded the ±0.75 threshold in MV-GWAS strategy. However, this approach exclusively identified a novel locus ATF3 with generally lower *P*-values compare to the standard univariate and PC-GWAS methods.

Though the PC-GWAS approach captured well the variation across FBC traits simultaneously in this study and identified more novel loci compare with the other two methods, it cannot be a replacement for both the standard univariate GWAS and MV-GWAS because there are still a number of known loci that were not identified by PC-GWAS in our study but were identified in the standard univariate GWAS(e.g., *R3HDM1*, *TPM4*).

To demonstrate the strength of these multivariate GWAS methods when used to complement each other, we collectively identified six novel loci (ANKRD26, PDZRN4, COL1A1, OTOR, TTLL11, ATF3) subject to replication and both methods also identified three known association loci (*HBB*, *ITFG3*, *HLA-C*). The multivariate methods evidence that multivariate genotype-phenotype method increase power and thus identify novel genotype-phenotype associations not found with univariate GWAS in the same dataset. Though the MV-GWAS improves *P*-value much better, the PC-GWAS strategy found more novel loci.

These multivariate methods could maximize novel loci discovery for other correlated phenotypes, such as lipid traits, liver function, cancers, anthropometry, immune disease, and others and might help to speed up drug discovery across a range of Cardiometabolic traits as previous studies have shown that FBC may serve as markers of proinflammatory state of metabolic syndrome and promoter of atherosclerotic risk (Jesri et al., 2005; Kotani et al., 2007; Kelishadi et al., 2010).

## Ethics Statement

This study was approved by the Science and Ethics Committee of the UVRI, the Ugandan National Council for Science and Technology, and the East of England-Cambridge South NHS Research Ethics Committee United Kingdom.

## Author Contributions

SF, DG, MS, and PK designed the study. SF performed the analyses. TC carried out the quality control and imputation. DG, MS, and PK directed the project. SF and ON wrote the manuscript. All authors contributed to the interpretation of the results and writing the article.

## Conflict of Interest Statement

The authors declare that the research was conducted in the absence of any commercial or financial relationships that could be construed as a potential conflict of interest.
