# Supplementary material for: Complimentary Methods for Multivariate Genome-Wide Association Study Identify New Susceptibility Genes for Blood Cell Traits
Source: Front Genet. 2019 Apr 26;10:334. doi: 10.3389/fgene.2019.00334 (PMC6497788; doi:10.3389/fgene.2019.00334)
Supplement: Supplementary file 1 [file Data_Sheet_1.docx]

*Supplementary figures and tables*


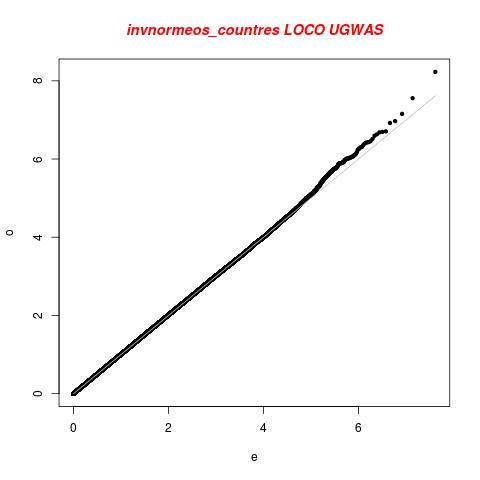

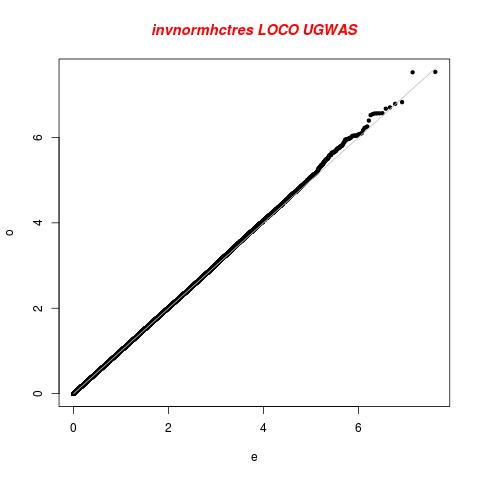

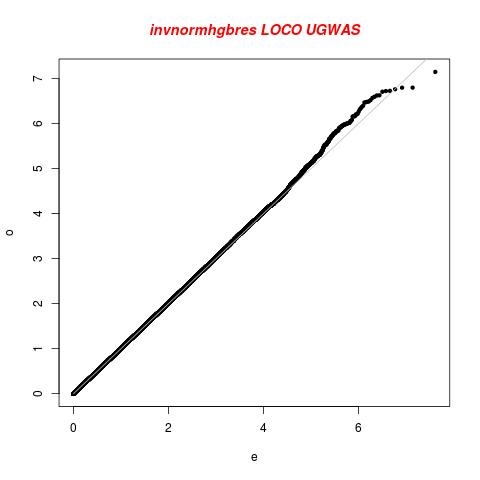

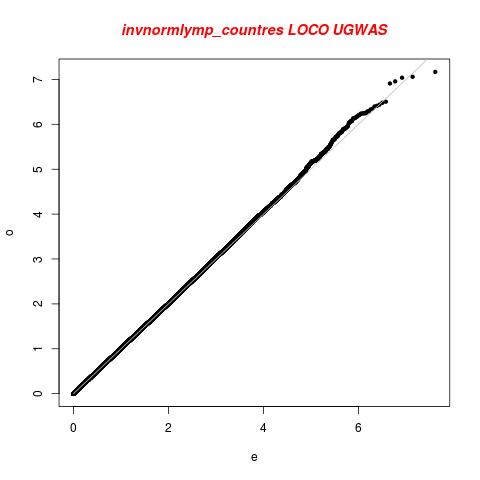

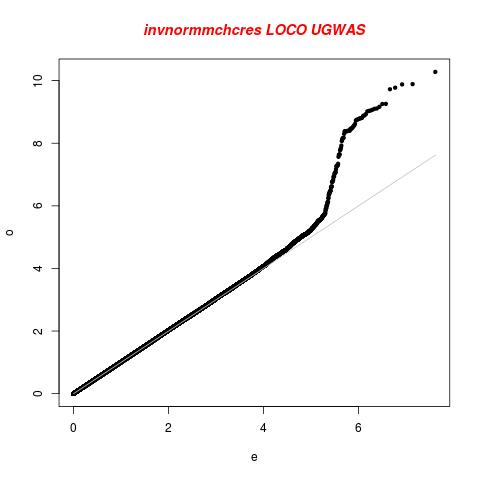

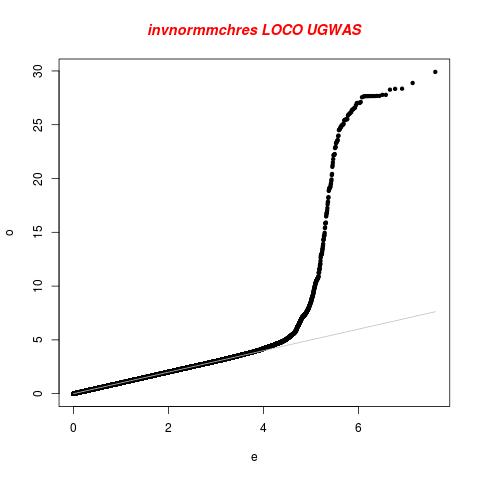

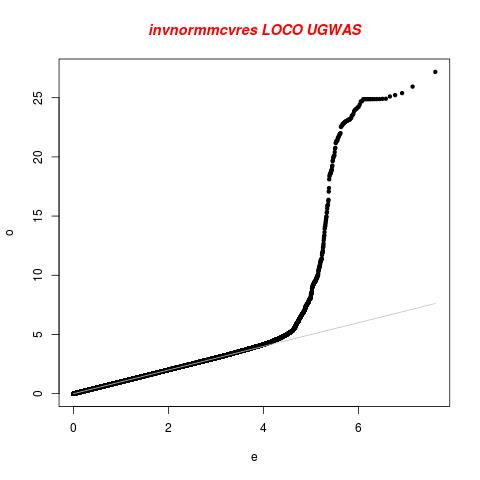

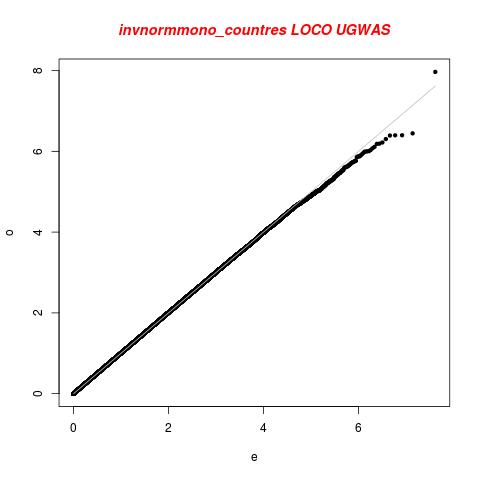

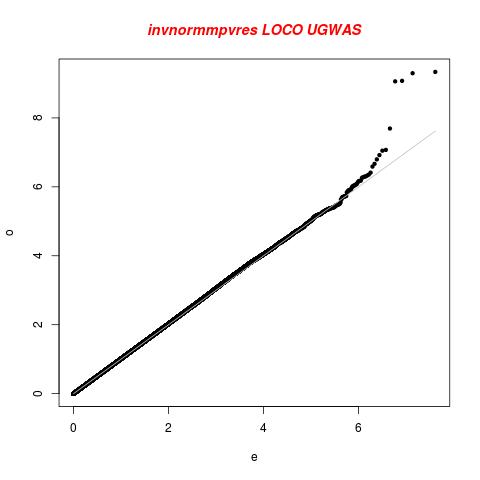

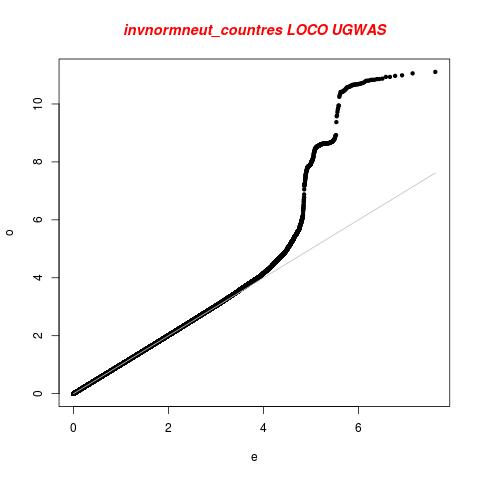

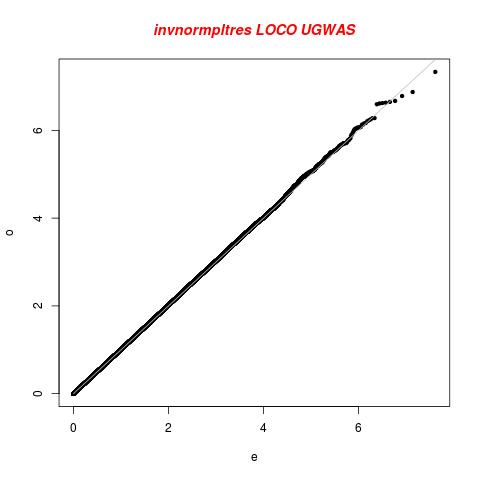

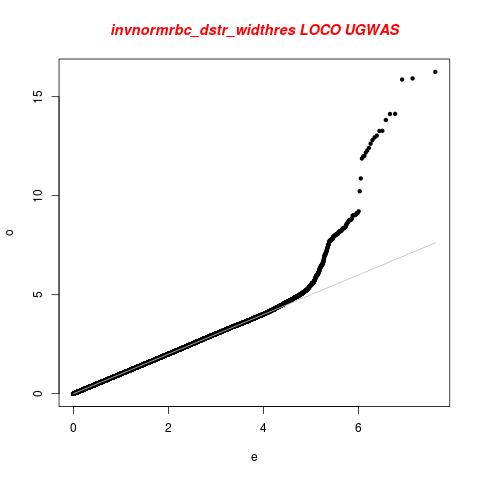

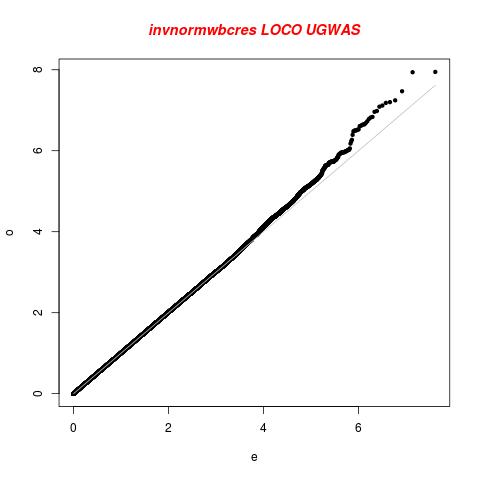

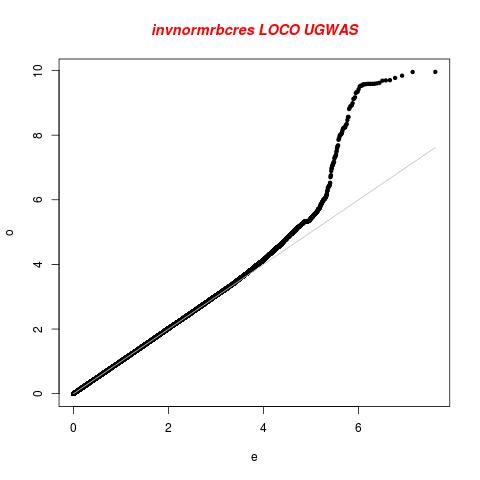


**Supplementary Figure 1: QQ plots of fifteen FBC traits analysed individually (univariate) showing** **the distribution of association P-values**


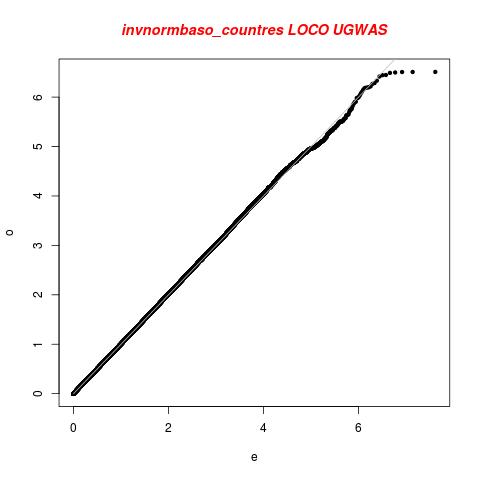


|  | **WBC** | **NEU** | **EOS** | **BASO** | **MONO** | **LYMPH** | **RBC** | **HGB** | **HCT** | **MCV** | **MCH** | **MCHC** | **RDW** | **PLT** | **MPV** |
| --- | --- | --- | --- | --- | --- | --- | --- | --- | --- | --- | --- | --- | --- | --- | --- |
| **WBC** | 1 | 0.08 | 0.1 | 0.17 | -0.17 | -0.17 | 0.14 | 0.11 | 0.14 | -0.03 | -0.07 | -0.11 | -0.01 | -0 | -0.03 |
| **NEU** | 0.08 | 1 | -0.36 | -0.19 | 0.05 | **-0.78** | -0.06 | -0.11 | -0.11 | -0.05 | -0.04 | 0.01 | 0.08 | -0 | -0.01 |
| **EOS** | 0.1 | -0.4 | 1 | 0.06 | -0.19 | -0.17 | 0.02 | 0.02 | 0.04 | 0.02 | 0 | -0.04 | -0.05 | -0 | -0.04 |
| **BASO** | 0.17 | -0.2 | 0.06 | 1 | -0.02 | 0.13 | 0.19 | 0.2 | 0.27 | 0.05 | -0.03 | -0.19 | -0.03 | -0 | -0.04 |
| **MONO** | -0.17 | 0.05 | -0.19 | -0.02 | 1 | -0.07 | -0.07 | -0.08 | -0.08 | 0 | 0 | -0.01 | -0.02 | 0 | 0.02 |
| **LYMPH** | -0.17 | **-0.8** | -0.17 | 0.13 | -0.07 | 1 | 0.07 | 0.1 | 0.09 | 0.02 | 0.02 | 0.02 | -0.05 | 0 | 0.03 |
| **RBC** | 0.14 | -0.1 | 0.02 | 0.19 | -0.07 | 0.07 | 1 | 0.67 | **0.75** | -0.55 | -0.54 | -0.17 | 0.07 | 0.1 | 0.13 |
| **HGB** | 0.11 | -0.1 | 0.02 | 0.2 | -0.08 | 0.1 | 0.67 | 1 | **0.94** | 0.16 | 0.24 | 0.27 | -0.29 | 0 | 0.03 |
| **HCT** | 0.14 | -0.1 | 0.04 | 0.27 | -0.08 | 0.09 | **0.75** | **0.94** | 1 | 0.12 | 0.07 | -0.06 | -0.29 | 0 | 0.04 |
| **MCV** | -0.03 | -0.1 | 0.02 | 0.05 | 0 | 0.02 | -0.55 | 0.16 | 0.12 | 1 | **0.92** | 0.18 | -0.46 | -0.1 | -0.13 |
| **MCH** | -0.07 | -0 | 0 | -0.03 | 0 | 0.02 | -0.54 | 0.24 | 0.07 | **0.92** | 1 | 0.53 | -0.41 | -0.1 | -0.13 |
| **MCHC** | -0.11 | 0.01 | -0.04 | -0.19 | -0.01 | 0.02 | -0.17 | 0.27 | -0.06 | 0.18 | 0.53 | 1 | -0.07 | -0 | -0.03 |
| **RDW** | -0.01 | 0.08 | -0.05 | -0.03 | -0.02 | -0.05 | 0.07 | -0.29 | -0.29 | -0.46 | -0.41 | -0.07 | 1 | 0.1 | 0.05 |
| **PLT** | -0.03 | -0 | -0.04 | -0.04 | 0.02 | 0.03 | 0.13 | 0.03 | 0.04 | -0.13 | -0.13 | -0.03 | 0.05 | 1 | -0.5 |
| **MPV** | -0.03 | -0 | -0.04 | -0.04 | 0.02 | 0.03 | 0.13 | 0.03 | 0.04 | -0.13 | -0.13 | -0.03 | 0.05 | -0.5 | 1 |

**Supplementary Table 1a: Correlation coefficient of all Full Blood traits in UGWAS**. **Correlations above +/-0.75 are highlighted in red**

| **Supplementary Table 1b: Description of highly correlated traits** | | |
| --- | --- | --- |
| **Trait 1** | **Trait 2** | **Correlation coefficient (*r2*)** |
| HGB | PCV | 0.94 |
| MCH | MCV | 0.92 |
| PCV | RBC | 0.75 |
| LYN | NEU | -0.78 |

| **Supplementary Table 2: Description of genomic infation factor and proportion of variance for each PC** | | | |
| --- | --- | --- | --- |
|  | **PCA** | **Genomic inflation factor** | **Proportion of variance** |
|  | PCA1 | 1.00 | 0.2342 |
|  | PCA2 | 1.01 | 0.1805 |
|  | PCA3 | 1.01 | 0.1631 |
|  | PCA4 | 1.01 | 0.09376 |
|  | PCA5 | 1.02 | 0.07265 |
|  | PCA6 | 1.01 | 0.06573 |
|  | PCA7 | 1.01 | 0.0514 |
|  | PCA8 | 1.01 | 0.04288 |
|  | PCA9 | 1.01 | 0.03695 |
|  | PCA10 | 1.01 | 0.02877 |
|  | PCA11 | 1.00 | 0.02652 |
|  | PCA12 | 1.02 | 0.00175 |
|  | PCA13 | 1.01 | 0.00118 |
|  | PCA14 | 1.00 | 0.00041 |
|  | PCA15 | 1.00 | 0.00025 |


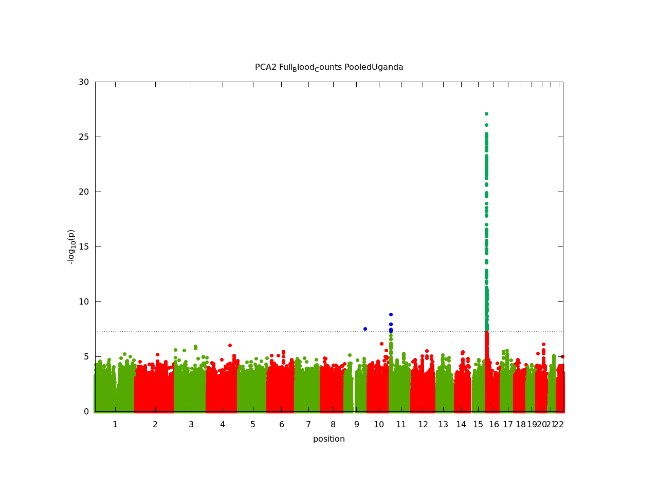

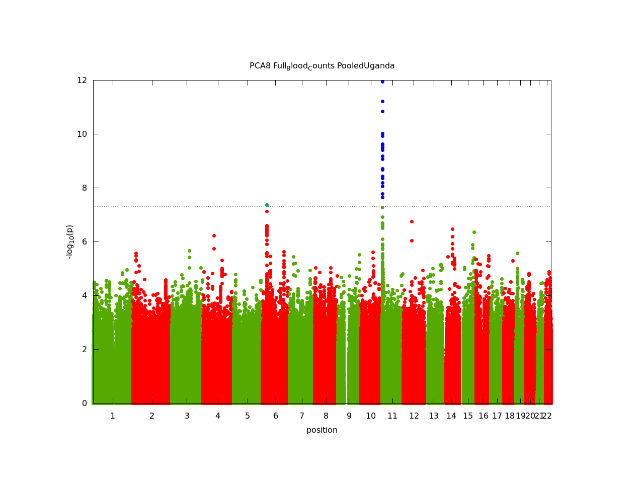

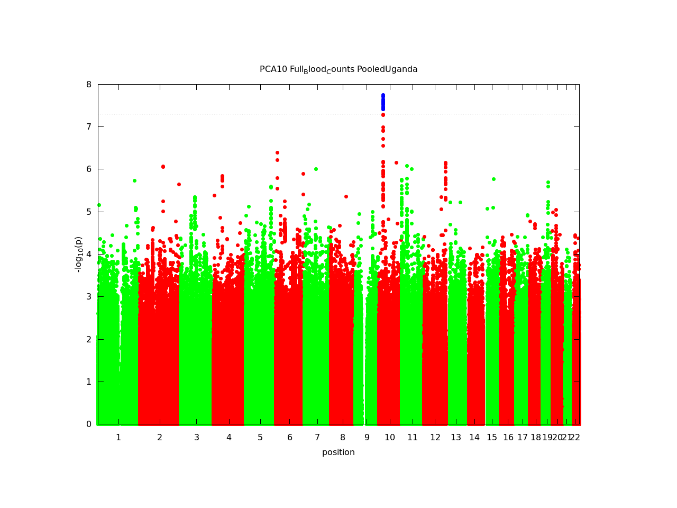

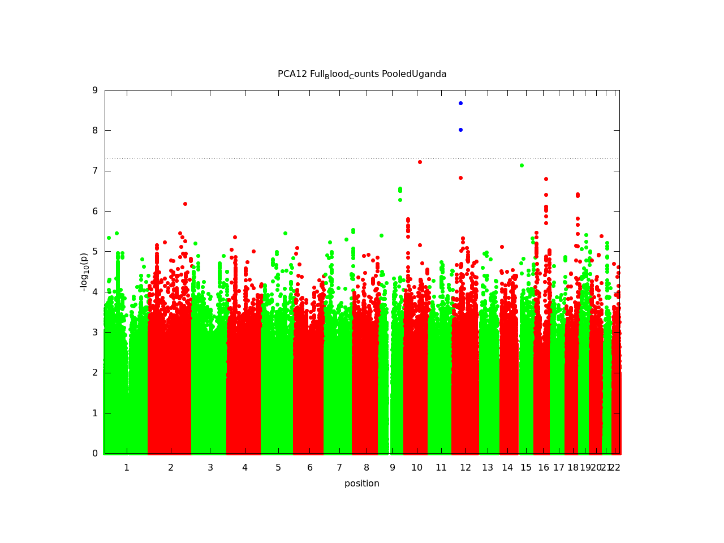

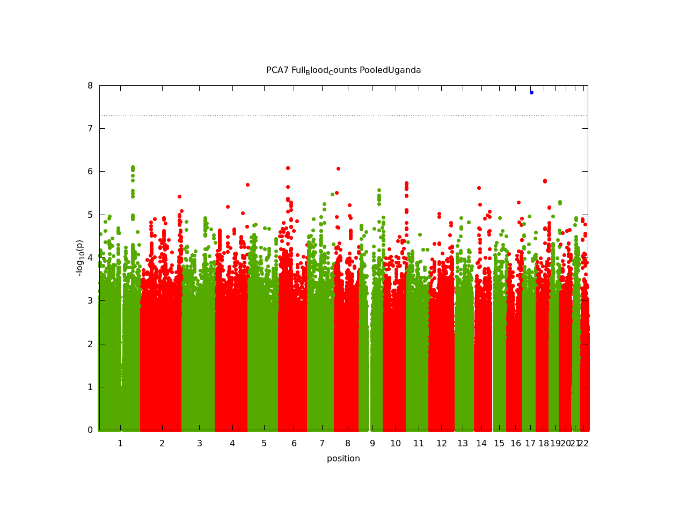

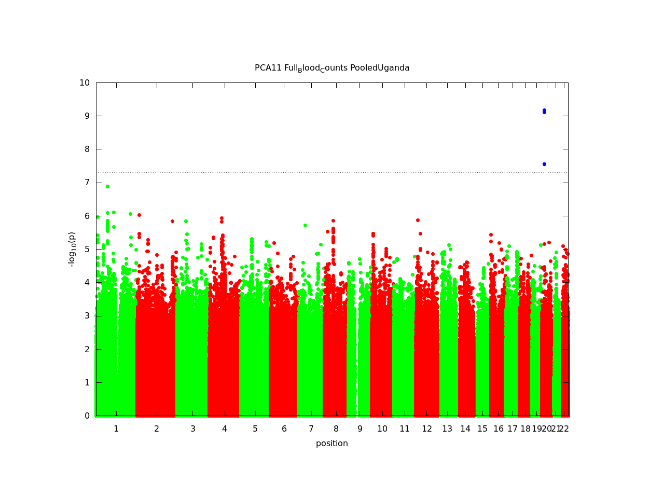


**Supplementary Figure 2: Manhattan plots of genome-wide associations using the PC-GWAS approach. Manhattan plots shown here have at least one genome-wide significant variant. At Principal Components 2, 7, 8, 10, 11, 12.**

PC 8

PC 7

PC 2

PC 12

PC 11

PC 10
